# Rational design of an acridine-derived click chemistry-based artificial metallo-nuclease

**DOI:** 10.1039/d6cb00097e

**Published:** 2026-06-15

**Authors:** Oliver Gould, Alex Gibney, Rebecca Lynn, Simon Poole, Bríonna McGorman, Andrew Kellett

**Affiliations:** a School of Chemical Sciences, Dublin City University, Glasnevin, Dublin 9 Dublin Ireland andrew.kellett@dcu.ie

## Abstract

Artificial metallo-nucleases (AMNs) are metal complexes capable of cleaving nucleic acids and represent a promising therapeutic class. We recently established that copper(i)-catalysed azide–alkyne cycloaddition (CuAAC) click chemistry offers a versatile approach for building new minor groove targeting AMNs, epitomised by the tri-click (TC) series; here, three bidentate chelation sites comprising the *N*-triazole donor from the CuAAC reaction, together with the ‘clicked’ donor group, provide new ligand architectures that coordinate up to three bioactive copper(ii) metal ions. Although the TC series are promising scaffolds, no route has yet been established to direct, or enhance their DNA recognition properties. Herein, we report a new method for hybridising click chemistry-based AMNs with acridine, a potent DNA intercalating agent. Motivation for generating this conjugate stems from the opportunity to combine multimodal DNA binding properties, namely, DNA intercalation *via* the acridine unit, and minor groove recognition and cleavage by the nuclease component. Two sites of the original TC scaffold were retained for copper chelation and DNA cleavage, thereby producing a di-click-pyridine (DC-Py) unit, while the third site was repurposed for conjugation to the acridine (A) group. The resultant hybrid (DC-PyA) was then coordinated with copper(ii) ions (Cu_2_-DC-PyA) and the downstream direct and indirect DNA recognition properties and cleavage properties were examined, and activity resembling threading DNA intercalation was observed.

## Abstract 2

Conjugation of an acridine anchor to a click chemistry-generated chemical nuclease yields Cu_2_-DC-PyA with multimodal DNA recognition, combining intercalation with minor groove binding and potent oxidative cleavage activity.

## Graphical abstract

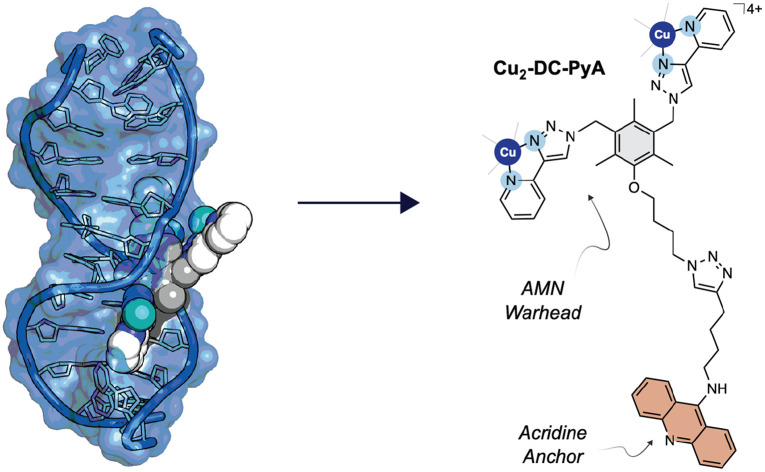

## Introduction

**Fig. 1:**
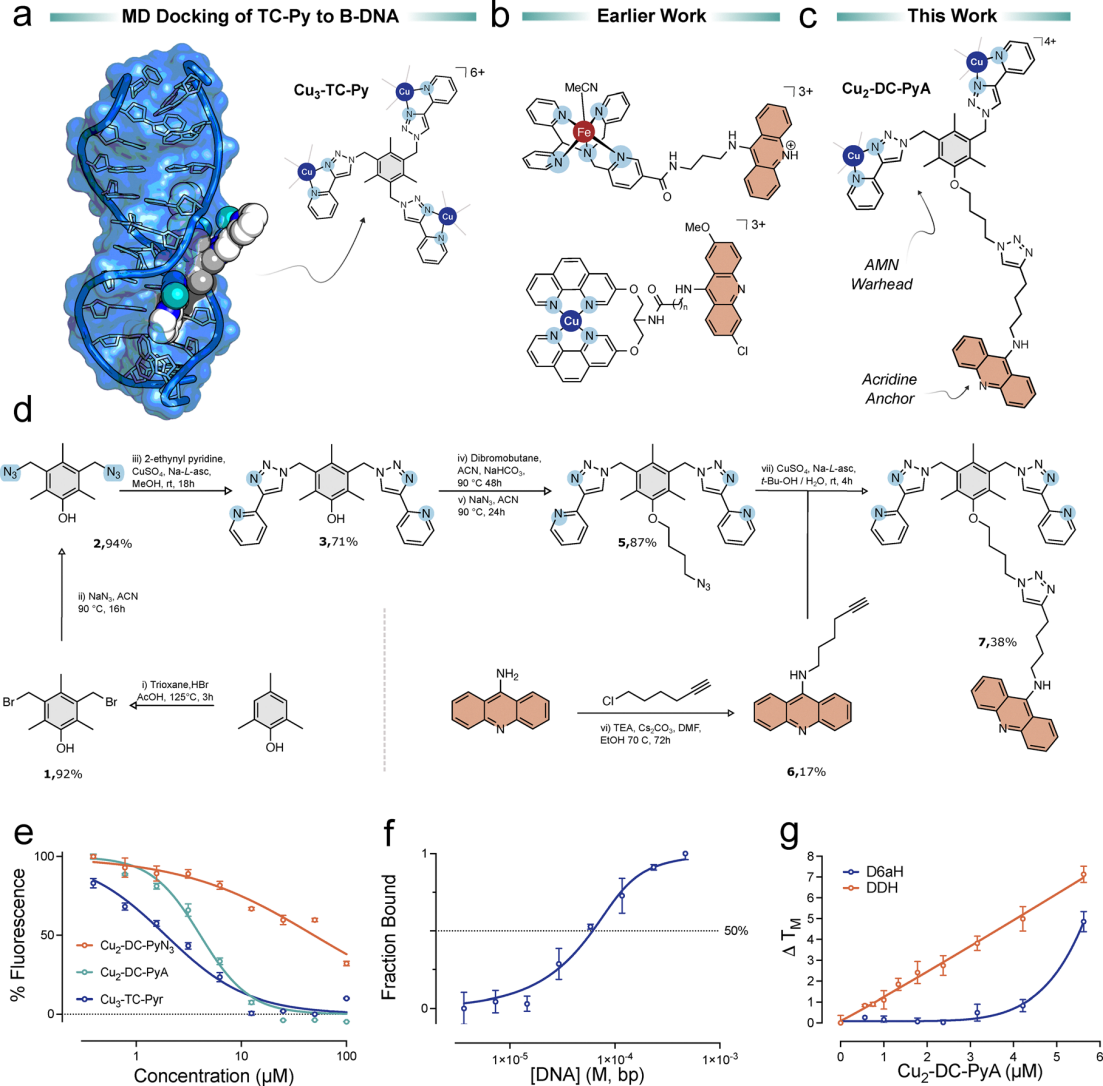
(a) Previously reported Cu_3_-TC-Py binds duplex DNA with an exposed “arm”. (b) Examples of AMNs that have shown enhanced activity upon conjugation to acridines. (c) Structure prepared in this study, Cu_2_-DC-PyA, a derivative of Cu_3_-TC-Py where one arm has been replaced with an acridine anchor. (d) Synthetic procedure for the synthesis of DC-PyA. (e) Fluorescent intercalator displacement assays enabled calculation of apparent binding constants. Cu_2_-DC-PyA shows enhanced binding affinity relative to its synthetic precursor Cu_2_-DC-PyN_3_ and similar affinity to the parent, Cu_3_-TC-Py. (f) Direct binding monitored by fluorescence upon titration of ctDNA, which enabled calculation of the direct binding constant of Cu_2_-DC-PyA using the Bard model. (g) Thermal melting analysis using FRET-modified DNA hairpins showed clear sequence discrimination. Non-linear regression included for presentation purposes only (concentration 10 µM and 7.5 µM were excluded due to condensation). All experiments were conducted in triplicate; all error bars indicate standard error of three values.

There has been a sustained interest in the development of new artificial metallo-nucleases (AMNs),^1–6^ in-part owing to the clinical success of bleomycin—an antineoplastic antibiotic that chelates Fe(ii), binds to DNA, and promotes oxidative DNA damage through DNA-localised Fenton-type chemistry.^7–9^ Recently, we reported a new strategy for AMN development using click chemistry, whereby the 1,2,3-triazole group formed during the copper(i)-catalysed azide–alkyne cycloaddition (CuAAC) reaction, together with proximal secondary alkynyl donors, chelates with up to three Cu(ii) ions to form trinuclear ‘tri-click’ (TC) complexes.^10–12^ Our most recent contribution in this area identified TC-Py (where Py = pyridine) as the most active AMN from a focused series of alkynyl donors with potential copper(ii) ion chelating properties.^12^ Mechanistic experiments involving molecular dynamics identified that two of the three triazole–Py arms in the Cu_3_-TC-Py complex actively participate in DNA binding (Fig. 1a), while the third arm does not directly contact the DNA surface. Although the third site likely enhances binding opportunities through its dynamic motion, its remote positioning suggests a rational choice for incorporating a directing group. While gene-targeting vectors like triplex-forming oligonucleotides offer significant opportunities for sequence-specific targeting,^13–15^ recent reviews highlight that conjugation of AMNs to established DNA-binding small molecules provides a direct method for AMN activity enhancement.^1,16^ Acridine and its analogues have been used to this end by successfully enhancing the nuclease activity of various AMNs (Fig. 1b),^17–21^ and have further promoted nuclear localisation of Au(i) and ^99m^Tc(I) complexes.^22,23^ Here, we report Cu_2_-DC-PyA (Fig. 1c), a TC-inspired, click-assembled hybrid, in which two Py-supported Cu(ii) centres are retained for minor-groove cleavage while the third position is repurposed as an acridine intercalator, enabling cooperative copper-mediated nuclease activity and dual-mode DNA recognition.

## Results and discussion

### Synthesis

DC-PyA was prepared using multi-step synthesis outlined in Fig. 1d. In order to generate a *C*_2_-symmetric analogue of the recently reported TC-Py, we started by alkylating mesitol to generate 1, prior to the installation of two azide handles in 2 by nucleophilic substitution with sodium azide.

Subsequent CuAAC with 2-ethynyl pyridine then produced 3, and the phenolic group allowed the incorporation of an alkynyl linker in 4*via* Williamson ether synthesis using excess dibromobutane. Azidation of the resulting alkyl halide was next performed with sodium azide to generate DC-PyN_3_ (5). In parallel, 9-amino acridine was alkylated *via* nucleophilic substitution to produce 9-hexynyl acridine (6). DC-PyN_3_ and 9-hexynyl acridine were then combined in a second CuAAC reaction to provide the final ligand, DC-PyA (7). The seven-step, synthetic workflow provided a final product yield of 20% from the mesitylene starting material, predominantly diminished by the final CuAAC step with 5 and 6, which required chromatographic purification. The attempted synthesis of 6 under standard nucleophilic substitution conditions, did not proceed (data not shown) and the reaction required use of caesium carbonate to promote formation of the desired intermediate, which showed poor chromatographic separation under flash conditions.^24^ All synthetic targets were characterised by ^1^H and ^13^C NMR. Azides 2 and 5 were additionally characterised by Fourier Transform Infrared (FT-IR) spectroscopy and liquid chromatography-mass spectrometry (LC-MS) was carried out on the final product (Fig. S1–S12).

### DNA binding studies

#### Apparent binding constants

Metal complex stocks were prepared *in situ* by mixing DC-PyA and two molar equivalents of copper(ii) nitrate trihydrate to produce Cu_2_-DC-PyA. The DNA binding affinity of Cu_2_-DC-PyA was initially evaluated using an ethidium bromide (EtBr)-based fluorescent intercalator displacement (FID) assay. Titration of a test compound into solutions of EtBr-saturated DNA results in competition and concentration-dependent depletion of EtBr fluorescence. Data from this assay can then be used to calculate an apparent DNA binding constant (*K*_app_) using eqn 1, where *K*_b_ is the equilibrium binding constant of EtBr, [EtBr] is the concentration of EtBr in the sample and *C*_50_ is the concentration of titrant required to reduce fluorescence by 50%.^25^1
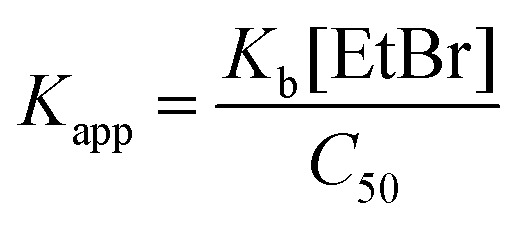


It is worth noting that FID assays are limited by their indirect nature and are affected by the *K*_b_ value used in eqn (1) along other factors such as temperature, sequence composition, and buffer. It is also important that the same *K*_b_ value and experimental conditions are used between experiments if *K*_app_ values are to be compared. Accordingly, we used a *K*_b_ value of 8.8 × 10^6^ M^−1^ and experimental conditions as recently reported^12^ to allow comparison to Cu_3_-TC-Py. Here, Cu_2_-DC-PyA returned a *K*_app_ value of 2.71 × 10^7^ M^−1^, similar to the value of 5.8 × 10^7^ M^−1^ previously reported for Cu_3_-TC-Py and exceeding that of other TC complexes.^10–12^ Interestingly, we found the *K*_app_ value of the target Cu_2_-DC-PyA was an order of magnitude higher than the azide precursor Cu_2_-DC-PyN_3_ (*K*_app_ = 2.2 × 10^6^ M^−1^), which lacks the conjugated acridine unit.

#### Direct DNA binding analysis

FID assays are useful tools for the analysis of compounds with otherwise unsuitable spectroscopic properties, such as those lacking a chromophore or presenting a dominant spectral overlap with DNA.^26^ However, the incorporation of acridine into Cu_2_-DC-PyA allowed us to directly monitor DNA binding of this unit by fluorescence spectroscopy (Fig. S13). Here, ctDNA was titrated into a buffered solution of Cu_2_-DC-PyA (10 µM; 80 mM HEPES, 25 mM NaCl, pH 7.4), and fluorescence changes were monitored at *λ*_ex_ = 415 nm and *λ*_em_ = 455 nm (Fig. S14). Results showed a clear increase in fluorescence with increasing DNA concentrations (Fig. S15 and S16). Data were modelled using the Bard equation (eqn (2) and (3))^27,28^ which best fit the data with a binding affinity of 6.5 × 10^5^ M^−1^ and a stoichiometry of 5 bp per Cu_2_-DC-PyA. Interestingly, this binding stoichiometry is lower than might be expected for a bifunctional molecule such as Cu_2_-DC-PyA and may indicate a cooperative binding mode. The lower direct binding constant relative to *K*_app_ likely reflects the fact that only the acridine component contributes to the fluorescence signal in direct measurements. Since the dicopper unit lacks a chromophore and is therefore spectroscopically silent under these conditions, binding events mediated solely by the Cu_2_-DC-Py component, through minor groove recognition in the absence of acridine intercalation are therefore not captured.

#### Thermal melting studies

Next, we evaluated the impact of Cu_2_-DC-PyA binding on the thermodynamic stability of hairpin DNA targets using a FRET melting method (Fig. 1g and Fig. S17–S19).^2^ Here, hairpin DNA constructs were modified to contain FRET partners of 3′-Iowa Black® and 5′-Alexa Fluor™ 647. When annealed the proximity of the FRET pair results in fluorescence quenching, and when denatured, the FRET pair separate to give a fluorescence signal.^29^

The thermal denaturation of the hairpin can therefore be monitored through the fluorescence signal and the midpoint of the melting curve identified as the melting temperature (*T*_m_).^30–32^ To investigate the sequence selectivity of Cu_2_-DC-PyA we monitored thermal melting changes (Δ*T*_m_) using two hairpin sequences containing varying GC content: DDH, which contains 66% GC content and D6ah, which contains 33% GC content.

Here, we found that Cu_2_-DC-PyA showed clear linear stabilisation of the DDH hairpin with an Δ*T*_m_ of ∼5 °C upon exposure to 5 µM of the complex. In contrast, the D6aH hairpin was minimally stabilised with a maximum Δ*T*_m_ of < 1.5 °C at the highest complex exposure. These results indicate that incorporation of the acridine moiety into Cu_2_-DC-PyA enhances the GC-sequence selectivity, likely reflecting an additive effect of GC-selectivity by 9-aminoacridine derivatives.^33^ This enhanced GC selectivity supports the intended intercalative role of the acridine unit suggesting the acridine motif engages DNA as designed.

#### Viscosity studies

A key feature of intercalation is the extension of the DNA double helix as the intercalator participates in π-stacking interactions with successive nucleobases.^34^ Consequently, methods sensitive to measuring hydrodynamic changes can be used to characterise DNA intercalation with viscosity being one of the most readily accessible methods.^35^ To validate intercalation by the acridine moiety in Cu_2_-DC-PyA we conducted relative viscosity experiments with stDNA (Fig. S20). The Cu-free ligand, DC-PyA (7) showed a clear concentration-dependent increase in relative viscosity in line with the trend observed for established intercalators EtBr and actinomycin D. In contrast, the di-nuclear complex Cu_2_-DC-PyA demonstrated a concentration-dependent decrease in relative viscosity, although this trend was less pronounced than that observed for the parent Cu_3_-TC-Py. Data here support the additive bifunctional binding mode suggested by *T*_m_ studies. Overall, the observed decrease in viscosity associated with DNA condensation appears due to the electrostatic groove binding properties of the di-copper(ii) component overriding the lengthening properties associated with acridine intercalation.

#### Topoisomerase inhibition

**Fig. 2:**
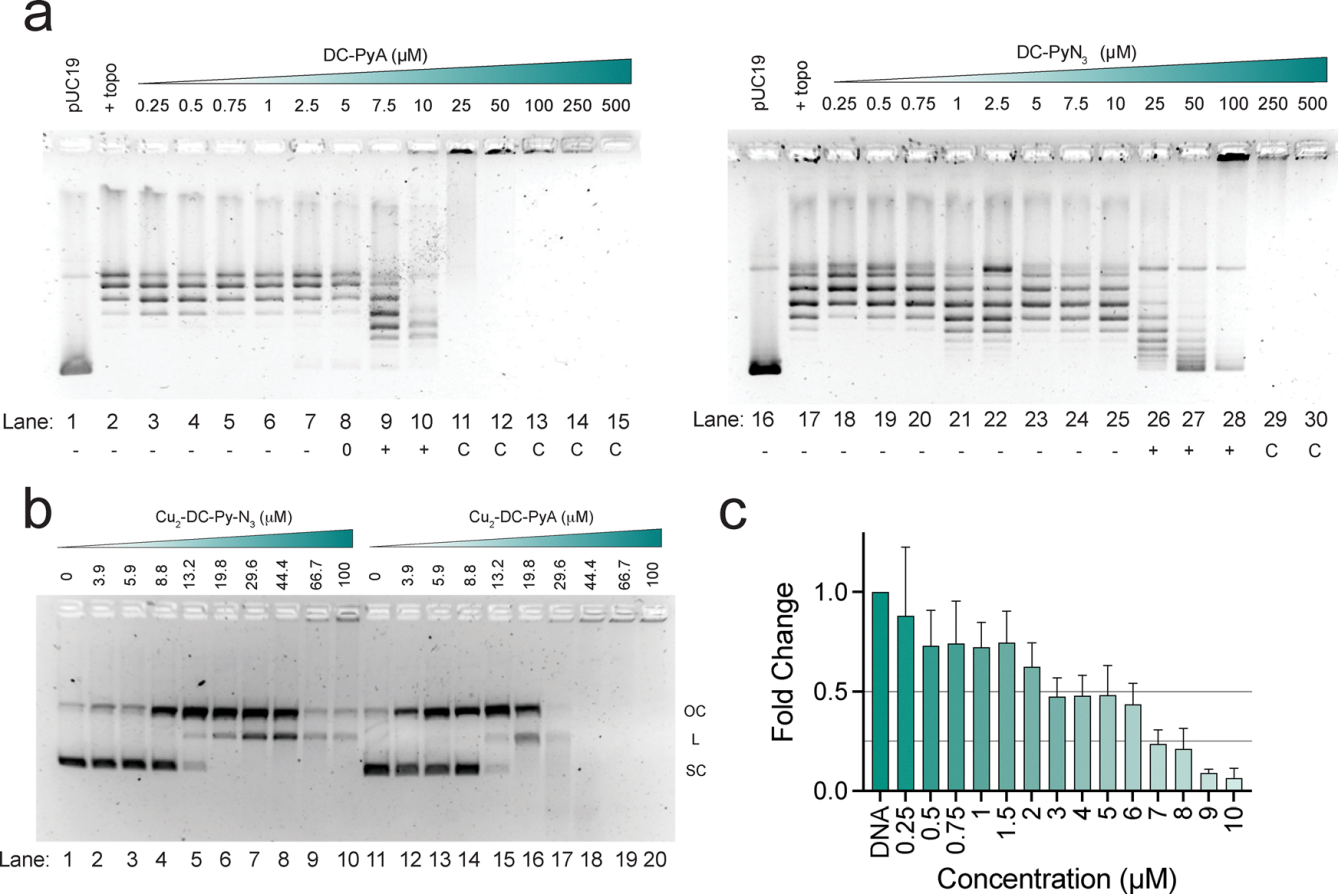
(a) Release of topological tension from supercoiled plasmid DNA using the topoisomerase I-mediated relaxation assay in the presence of increasing concentrations of Cu_2_-DC-PyA (left) and Cu_2_-DC-PyN_3_ (right). (b) Supercoiled pUC19 cleavage profile of Cu_2_-DC-PyN_3_ (lanes 1–10) and Cu_2_-DC-PyA (lanes 11–20). (c) Relative quantification of cleavage induced by Cu_2_-DC-PyA.

To further investigate the intercalative properties of Cu_2_-DC-PyA, a topoisomerase IA (Topo) mediated relaxation assay was performed.^36^ Topo enzymes play an important role in regulating genome stability by relieving DNA topological constraints generated during processes such as gene transcription, DNA replication, recombination, and repair.^37^ This is achieved through formation of a transient break in the DNA backbone, which permits topological relaxation, prior to strand ligation.^38^ Unwinding of negatively supercoiled (SC) pUC19 DNA by Cu_2_-DC-PyA was examined and compared to the non-acridine containing precursor, Cu_2_-DC-PyN_3_ (Fig. 2a). Relaxation of the plasmid to the open circular form (0) was identified upon exposure to 5 µM of Cu_2_-DC-PyA (lane 8). Higher concentrations of the complex (7.5 and 10 µM; lanes 9 and 10) generated positively (+) supercoiled DNA, beyond which the plasmid underwent condensation (lanes 11–15) presumably due to the high cationic charge borne by the hybrid. In contrast, Cu_2_-DC-PyN_3_ relaxed SC DNA between exposure values of 10 and 25 µM (lanes 10 and 11), indicating the acridine group plays an important role in enhancing the intercalating properties of the hybrid.

### Artificial metallo-nuclease activity

#### pUC19 relaxation assay

The artificial metallo-nuclease activity of Cu_2_-DC-PyA was initially evaluated using an electrophoretic mobility shift assay (EMSA).^39,40^ Here, supercoiled pUC19 plasmid DNA was incubated with increasing concentrations of the complex, together with the exogenous reductant Na-l-ascorbate, and the conversion of supercoiled (SC) DNA to open-circular (OC) and linear (L) DNA forms was analysed by agarose gel electrophoresis. To understand how the acridine group impacts chemical nuclease activity, the DNA damage profile of Cu_2_-DC-PyA was compared to Cu_2_-DC-PyN_3_ (Fig. 2b). Experiments with Cu_2_-DC-PyA showed formation of the nicked OC form at the lowest exposure (lane 12 : 2.0 µM), while the L form, which arises due to the formation of double strand breaks, emerged at 6.6 µM (lane 15), with complete ablation observed at concentrations >9.9 µM (lanes 17–20). Next, the cleavage profile of Cu_2_-DC-PyN_3_ was monitored under identical conditions (lanes 2–10). Initially, the cleavage profile was similar to that of the acridine hybrid, with OC and L forms observed at 2.0 µM (lane 2) and 6.6 µM (lane 5), respectively. However, at concentrations >9.9 µM (lanes 7–10), larger fractions of OC and L forms persist compared to Cu_2_-DC-PyA treated samples. Overall, although both complexes are active chemical nucleases, it is clear the acridine hybrid outperforms the azide precursor, particularly at elevated concentrations.

#### Real-time PCR

Next, to quantify the DNA damage observed in the relaxation assay, the nuclease activity of Cu_2_-DC-PyA was evaluated by real-time PCR (qPCR).^41–43^ Here, a 411 bp amplicon from pUC19 was incubated with increasing concentrations (0.25–10 µM) of Cu_2_-DC-PyA and Na-l-ascorbate at 37 °C for 30 min. Reactions were then quenched with EDTA and qPCR was performed and monitored in real time through SYBR Green I fluorescence. The threshold cycle (*C*_T_) was determined, and the fold change in intact DNA was calculated and normalised to a non-treated control (Fig. 2c). Significant damage was observed from 2 µM, with 50% of the amplicon depleted upon exposure to 3 µM of the complex. Finally, just 25% of the amplicon remained at 7 µM with smaller fractions remaining thereafter. Overall, these data corroborate Cu_2_-DC-PyA as an efficient artificial metallo-nuclease, capable of ablating DNA and preventing downstream processing by Taq DNA polymerase.

#### Groove blocking analysis

**Fig. 3:**
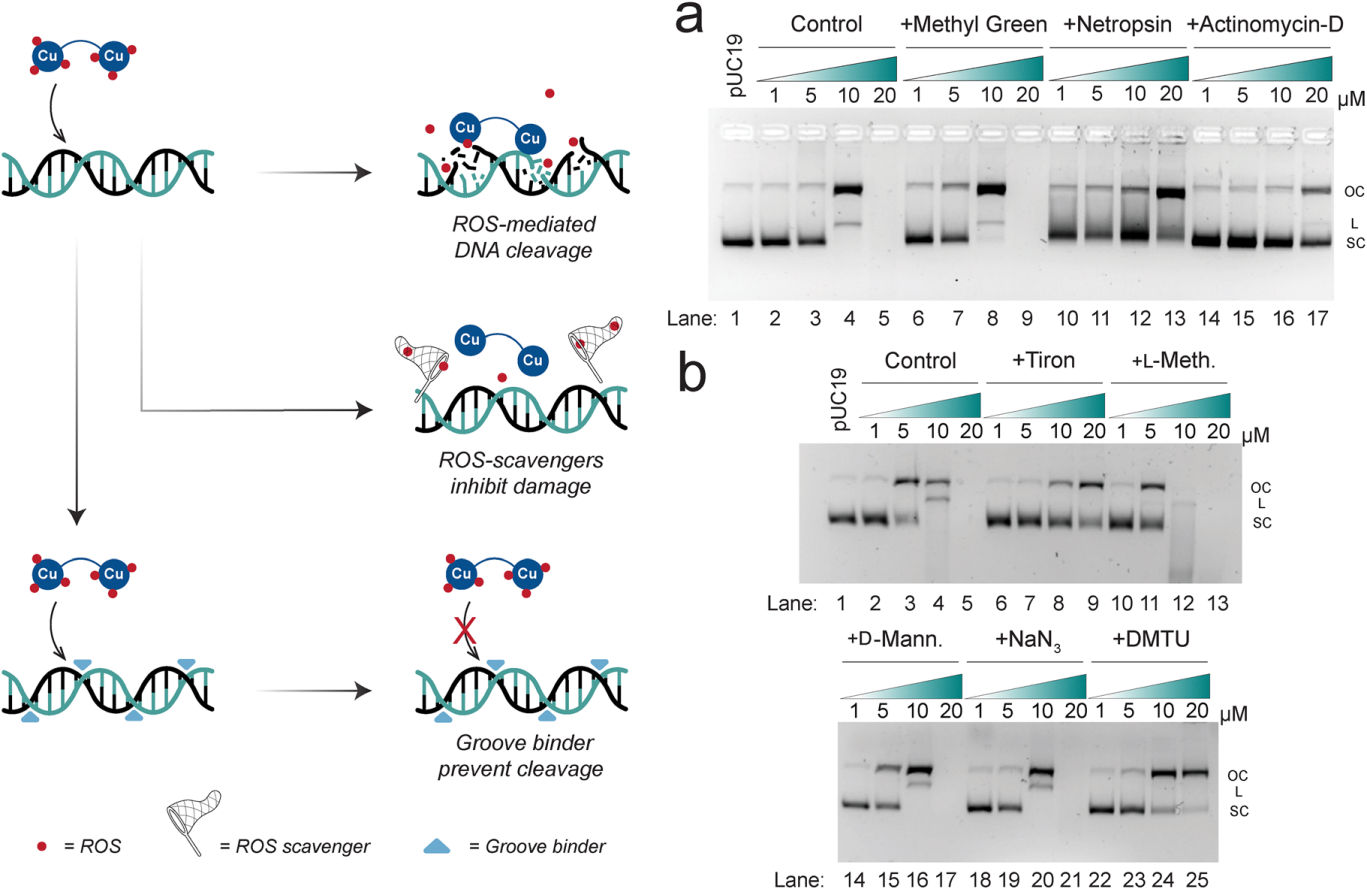
Cartoon representation of nuclease activity, and the impact of ROS scavengers and groove blocking groups. (a) Agarose gel analysis of the Cu_2_-DC-PyA (1, 5, 10 and 20 µM) cleavage of pUC19 (lanes 1–5) and Cu_2_-DC-PyA (1, 5, 10 and 20 µM) cleavage of pUC19 DNA that was pre-treated with methyl green (lanes 6–9), netropsin (lanes 10–13) and actinomycin-D (lanes 14–17). (b) Cleavage inflicted by Cu_2_-DC-PyA (1, 5, 10 and 20 µM) on supercoiled plasmid DNA (lanes 1–5) that was pre-incubated with ROS scavengers tiron (lanes 6-9), l-methionine (lanes 10–13), d-mannitol (lanes 14–17), NaN_3_ (lanes 18–21) and DMTU (lanes 22–25).

To probe the cleavage mechanism of Cu_2_-DC-PyA, assays were performed in the presence of competitive DNA binding agents (Fig. 3a and Fig. S21). Here, we employed methyl green and netropsin to probe the cleavage activity from the respective major and minor grooves,^44,45^ while EtBr probed competition for intercalative sites and ActD acted as a dual competitor for intercalative sites and minor groove occupancy.^46–49^ We observed that methyl green had no impact on cleavage activity while netropsin significantly inhibited cleavage by Cu_2_-DC-PyA, suggesting that cleavage primarily occurs from the minor groove, similar to the parent Cu_3_-TC-Py and earlier reported TC compounds. EtBr somewhat inhibited the activity while ActD was the most inhibitory competitor tested, with cleavage only observed at the highest tested concentration (20 µM). In total, these results further support the dual intercalative and minor groove binding modes suggested by thermal melting and viscosity studies and indicate Cu_2_-DC-PyA retains the minor groove regiospecific cleavage activity found for other TC complexes.

#### Scavenger studies

To determine the radical species involved in the Cu_2_-DC-PyA cleavage pathway, experiments were performed using reactive oxygen species (ROS) scavengers: Tiron (superoxide; O_2_˙^−^), l-methionine (hydroxyl radical; ˙OH, hypochlorous acid; HOCl, and hydrogen peroxide; H_2_O_2_); d-mannitol (hydroxyl radical; ˙OH); NaN_3_ (singlet oxygen, ^1^O_2_) and DMTU (hydrogen peroxide; H_2_O_2_ and the hydroxyl radical; ˙OH). Each ROS scavenger was added to pUC19 DNA prior to the addition of Cu_2_-DC-PyA and Na-l-ascorbate, and the cleavage profile was observed using the same EMSA protocol as in the initial cleavage analysis (Fig. 3b). A diminished cleavage profile was observed in the presence of tiron and DMTU, which implicates O_2_˙^−^, H_2_O_2_ and ˙OH as the primary ROS involved in the cleavage pathway. Interestingly, we observed enhanced DNA damage in the presence of l-methionine. This activity might be linked to enhanced reduction of copper(ii) by the thioether component of l-methionine, or that by sequestering both H_2_O_2_ and ˙OH, cleavage activity is activated through the more reactive O_2_˙^−^ pathway. This result is directly comparable to that of Cu_3_-TC-Py, which demonstrates the mechanism is conserved upon the replacement of one Py unit with the acridine group.

## Conclusions

Click chemistry has provided new avenues for the design and synthesis of metallodrugs, enabling the modular synthesis of artificial metallo-nucleases (AMNs) *via* a “click to chelate” strategy.^50,51^ However, only a limited number of compounds have used the triazole group generated from the CuAAC reaction with proximal secondary donors to chelate Cu(ii) ions. This concept was previously explored within our “tri-click” (TC) series, with recent work highlighting Cu_3_-TC-Py as a leading AMN.^10–12^ In the TC-Py study, molecular dynamics indicated that only two of the copper-chelating units in TC-Py could simultaneously bind with DNA, and in this work we exploited the opportunity to repurpose the third position with an acridine DNA intercalator. This resulted in the “Di-click” (DC-PyA) derivative, a *C*_2_-symmetric hybrid with augmented minor groove recognition and DNA intercalating properties. The primary aim of this work was to demonstrate that a significant change to the central modality of the TC series, reassembling one of the three arms to house a DNA-targeting intercalator with fluorescent properties could yield a new class of hybrid AMN. Ethidium bromide displacement assays revealed that Cu_2_-DC-PyA displayed similar DNA binding affinity to the previous Cu_3_-TC-Py and enhanced affinity compared to its azide-precursor (Cu_2_-DC-PyN_3_).

The binding profile of Cu_2_-DC-PyA was further evaluated through FRET thermal melting where we observed a significant selectivity for GC-rich DNA. This enhanced selectivity for GC-rich DNA, relative to the parent Cu_3_-TC-Py, indicated the acridine moiety was intercalating as intended and we further confirmed this in relative viscosity and topoisomerase I inhibition experiments. Viscosity experiments showed that the acridine group caused an increase in relative viscosity in the absence of Cu^2+^ ions and offset the decrease in the same relative to Cu_3_-TC-Py. Topoisomerase studies further validated the additive effect of acridine conjugation with a markedly enhanced Topo I inhibition profile relative to the azide precursor. The DNA damaging properties of Cu_2_-DC-PyA were assessed using a combination of qPCR and EMSA assays where again acridine conjugate showed significantly enhanced activity relative to the synthetic precursor. Mechanistic studies with ROS scavengers and competitive binders showed that Cu_2_-DC-PyA retained the molecular mechanism and regioselectivity of Cu_3_-TC-Py.

In the broader landscape of minor groove-directing hybrids, Cu_2_-DC-PyA provides an interesting extension to established conjugates, such as Fe-EDTA-distamycin,^52^ Cu-Phen-Hoechst-33258,^53^ and the Cu-clip-phen-spermine series,^54^ which utilise high-affinity binders to direct oxidative damage. However, the synthetic strategy reported here offers enhanced modularity and synthetic ease afforded by click chemistry. Furthermore, unlike traditional hybrids where the linker serves a purely structural role, the triazole moiety in DC-PyA is functionally integrated, serving both as a covalent bridge and as a donor group that chelates bioactive copper(ii) ion; this approach simplifies the preparation of new hybrids and allows for the diversification of the DNA binding or directing unit. Consequently, this study may serve as a gateway to access more sophisticated systems. In summary, by repurposing the third chelating arm of Cu_3_-TC-Py with a targeting unit, we have established a blueprint for generating future high-affinity, site-specific DNA damaging agents.

## Experimental

### General remarks

All synthesis was conducted under atmospheric conditions unless otherwise stated. Chemical reagents were sourced from Merck, Tokyo Chemical Industry (TCI) and Fluorochem unless otherwise stated and were used without any further purification. Biological reagents were purchased from New England Biolabs or FisherScientific unless otherwise indicated. HPLC grade chloroform and methanol were used without further purification. ^1^H and ^13^C NMR spectra were obtained on a Bruker AC 600 MHz NMR spectrometer and processed in MNova (MastreLab). FTIR was performed on a PerkinElmer Spectrum Two FTIR. LC-MS was performed on an Agilent Technologies 1200 Series instrument consisting of a G1322A Quaternary pump and a G1314B UV detector (254 nm) coupled to an Advion Expression L Compact Mass spectrometer (ESI) operating in positive mode. Fluorescence quenching assays were performed on a TECAN Spark® microplate reader. Agarose gel electrophoresis was performed using a Bio-Rad wide-cell mini sub system with a Bio-Rad basic Power Pac^TM^. All gels were imaged using a Syngene G:Box 9 mini gel documentation system. Real-time PCR (qPCR) and fluorescence thermal melting were performed on a Roche LightCycler 480 II.

### Synthesis

#### 3,5-bis(bromomethyl)-2,4,6-trimethylphenol – 1 (dibromide)

To a solution of trioxane (0.661 g, 7.34 mmol), HBr (in acetic acid) (20 mL) was added slowly. The reaction was heated to 75 °C until fully dissolved. To the reaction, mesitylene (1 g, 7.34 mmol) and H_2_O (0.833 mL) were added. The reaction was then refluxed at 125 °C for 3 h whilst stirring. The reaction was then allowed to cool to rt whilst stirring. Ice cold water was then added to the reaction flask to form the precipitate. Vacuum filtration was used to remove excess water and dry the product resulting in a light brown powder. Yield: 92%. ^1^H NMR (600 MHz, DMSO) *δ* 8.27 (s, 1H), 4.72 (s, 4H), 2.31 (s, 3H), 2.24 (s, 6H). Fig. S1.

#### 3,5-bis(azidomethyl)-2,4,6-trimethylphenol – 2 (diazide)

To a solution of 3,5-bis(bromomethyl)-2,4,6-trimethylphenol (2.16 g, 6.72 mmol) in MeCN (30 mL), sodium azide (4.37 g, 67.17 mmol) was added slowly in the reaction flask. This reaction was then refluxed at 90 °C for 24 h whilst stirring. The resulting product was then filtered, and solvents removed by rotary evaporation leaving a brown solid. Yield: 94%. ^1^H NMR (600 MHz, DMSO) *δ* 8.26 (s, 1H), 4.49 (s, 4H), 2.29 (s, 3H), 2.23 (s, 6H). FTIR: 2088 (s, N_3_) cm^−1^. Fig. S2 and S3.

#### 2,4,6-trimethyl-3,5-bis((4-(pyridin-2-yl)-1*H*-1,2,3-triazol-1-yl)methyl)phenol – 3 (DC-Py)

Diazide (1.53 g, 6.21 mmol) was dissolved in chloroform and methanol (1 : 1). Copper sulphate (0.050 g, 0.313 mmol) was dissolved in 1 mL of deionized water, and sodium ascorbate (0.100 g, 1.000 mmol) dissolved in 1 mL of deionized water. Copper sulphate solution was added dropwise to the sodium ascorbate solution (light blue – dark yellow/orange (reduction from copper(ii) to copper(i))). Copper sulphate-sodium ascorbate solution was added dropwise to the diazide solution and stirred until dissolved. Ethynylpyridine (1.28 g, 12.43 mmol) was dissolved in chloroform and methanol (1 : 1) and subsequently added dropwise to the reaction flask. Round bottom flask flushed with nitrogen gas. Bung added to flask and left to stir at RT for 72 h. Product was then filtered and dried to give a beige solid. Yield: 71%. ^1^H NMR (600 MHz, DMSO) *δ* 8.55 (s, 2H), 8.27 (s, 2H), 8.01 (s, 2H), 7.90 (s, 2H), 7.34 (s, 2H), 5.71 (s, 4H), 2.37 (s, 3H), 2.28 (s, 6H). Fig. S4.

#### 2,2′-(((5-(4-bromobutoxy)-2,4,6-trimethyl-1,3 phenylene) bis(methylene))bis (1*H*-1,2,3-triazole-1,4-diyl))dipyridine – 4 (DC-PyBr)

DC-Py (1.66 g, 3.67 mmol) was dissolved in acetonitrile (20 mL) (stirred with heat until dissolved), then sodium bicarbonate (3.08 g, 36.68 mmol) was added and dissolved. Subsequently, a solution of sodium hydroxide (0.09 g, 5.50 mmol) was added, followed by 1,4-dibromobutane (4.84 g, 22.01 mmol) in acetonitrile (20 mL). The reaction was then heated to 90 °C and refluxed and stirred overnight. The solution containing the product was then dried under rotary evaporation, dissolved in dichloromethane and washed with deionized water. It was then rinsed with portions of EDTA. The organic layer containing the product was then dried with magnesium sulphate. The resulting solution was then dried under rotary evaporation and then precipitated through dropwise addition to hexane solution and dried under vacuum. Yield: 28%. ^1^H NMR (600 MHz, DMSO) *δ* 8.55 (ddd, *J* = 4.9, 1.8, 1.0 Hz, 2H), 8.34 (s, 2H), 8.00 (d, *J* = 7.9 Hz, 2H), 7.88 (td, *J* = 7.6, 1.8 Hz, 2H), 7.33 (ddd, *J* = 7.5, 4.8, 1.2 Hz, 2H), 5.73 (s, 4H), 3.71 (t, *J* = 6.4 Hz, 2H), 3.63 (t, *J* = 6.7 Hz, 2H), 2.39 (s, 3H), 2.35 (s, 6H), 2.07–2.00 (m, 2H), 1.92–1.84 (m, 2H). Fig. S5.

#### 2,2′-(((5-(4-azidobutoxy)-2,4,6-trimethyl-1,3-phenylene)bis(methylene))bis(1*H*-1,2,3-triazole-1,4-diyl))dipyridine – 5 (DC-PyN_3_)

To a solution of DC-Py bromide (0.167 g, 0.285 mmol) in MeCN (20 mL), sodium azide (0.186 g, 2.85 mmol) was added slowly into the reaction flask. This reaction was then refluxed at 90 °C for 24 h whilst stirring. The resulting product was then filtered, and solvents were removed by rotary evaporation. Yield: 87%. ^1^H NMR (600 MHz, CD_3_CN) *δ* 8.51 (d, *J* = 4.0 Hz, 2H), 8.06 (d, *J* = 8.0 Hz, 4H), 7.83 (td, *J* = 7.8, 1.9 Hz, 2H), 7.29–7.27 (m, 2H), 5.69 (s, 4H), 3.73 (t, *J* = 6.3 Hz, 2H), 3.39 (t, *J* = 6.7 Hz, 2H), 2.39 (s, 3H), 2.35 (s, 6H), 1.89–1.77 (m, 4H). ^13^C NMR (151 MHz, DMSO) *δ* 150.26, 150.00, 147.44, 137.69, 132.60, 131.56, 130.65, 123.49, 123.17, 119.92, 119.87, 51.04, 49.00, 27.37, 25.69, 16.08, 13.47, 13.16. FTIR: 2094 (s, N_3_) cm^−1^. Fig. S6–S8.

#### *N*-(hex-5-yn-1-yl)acridin-9-amine – 6 (Hexynyl acridine)

9-Amino acridine (1.00 g, 5.15 mmol) and caesium carbonate (3.35 g, 10.30 mmol) were added to a round bottom flask with DMF and stirred until dissolved. 6-Chlorohexyne (3.00 g, 25.74 mmol) was then added with triethylamine (50 mL) and ethanol. The reaction was left to stir at 70 °C for 96 hours. It was then filtered, and the solvent was removed *in vacuo*. The crude product was purified by column chromatography to yield a yellow oil. Yield: 16.7%. ^1^H NMR (600 MHz, CDCl_3_) *δ*: 8.16 (d, 2H), 7.96 (d, 2H), 7.44 (d, 2H), 7.22 (t, 2H), 4.08 (t, 2H), 3.31 (d, 1H), 2.38 (t, 2H), 2.17 (t, 2H), 1.82 (t, 2H). Fig. S9.

#### *N*-(4-(1-(4-(2,4,6-trimethyl-3,5-bis((4-(pyridin-2-yl)-1*H*-1,2,3-triazol-1-yl)methyl) phenoxy) butyl)-1*H*-1,2,3-triazol-4-yl)butyl)acridin-9-amine – 7 (DC-PyA)

DC-PyN_3_ (0.258 g, 0.469 mmol) was dissolved in *tert*-butanol and water (1 : 1). Copper sulphate (0.852 g, 0.364 mmol) was dissolved in 1 mL of deionized water and sodium ascorbate (1.10 g, 0.555 mmol) was dissolved in 1 mL of deionized water. The copper sulphate solution was added dropwise to sodium ascorbate solution (light blue–dark yellow/orange) (reduction from copper(ii) to copper(i)). The copper sulphate–sodium ascorbate solution was added dropwise to the diazide solution and was stirred until dissolved. Hexynyl acridine (0.130 g, 0.469 mmol) was dissolved in *tert*-butanol and water (1 : 1) and was added dropwise to the reaction flask. The reaction was stirred at room temperature for 4 h. The product was then filtered and dried. The crude product was purified by column chromatography, to give a yellow powder. Yield: 37.7%. ^1^H NMR (600 MHz, (CD_3_)_2_SO) *δ*: 8.49 (d, 2H), 8.19 (m, 2H), 8.19 (m, 2H), 8.05 (d, 2H), 7.82 (d, 2H), 7.76 (m, 2H), 7.56 (s, 2H), 7.34 (s, 1H), 7.29 (t, 2H), 7.19 (t, 2H), 5.85 (s, 4H), 4.44 (t, 2H), 3.98 (t, 2H), 3.69 (t, 2H), 2.82 (t, 2H), 2.33 (s, 3H), 2.24 (s, 6H), 2.18 (m, 2H), 1.98 (m, 2H), 1.94 (m, 2H), 1.83 (m, 2H). ^13^C NMR (151 MHz, CDCl_3_) *δ* 153.73, 149.12, 148.13, 147.14, 146.31, 136.07, 132.83, 132.02, 131.97, 130.84, 129.39, 123.02, 121.99, 121.94, 120.21, 120.13, 120.09, 120.02, 119.32, 71.02, 49.00, 48.48, 48.03, 29.03, 26.25, 26.20, 26.14, 25.27, 23.86, 14.80, 11.99. ESI-MS: [M+H]^+^*m*/*z* calculated = 824.5, found = 824.5. Fig. S10–S12.

#### Fluorescence competition assay

EtBr displacement assays were carried out using methods previously reported. In 96 well plates, a serial dilution of each complex was prepared to a volume of 50 µL in 80 mM HEPES, 25 mM NaCl with 5% DMSO. To this, 50 µL of a working solution containing 25 µM EtBr and 25 µM ctDNA in the same buffer was added to give final conditions of 12.5 µM EtBr, 12.5 µM ctDNA and varying analyte concentration in a volume of 100 µL. Blank wells were prepared to contain no DNA and control wells contained no titrant/analyte. Plates were allowed to equilibrate for 30 min prior to fluorescence measurements using excitation at 530 nm and detection 590 nm. Resulting data was modelled using non-linear regression in GraphPad Prism and used to calculate *K*_app_ values as reported. Data plotted of TC-Py was obtained from the source data of previously reported *K*_app_ values of TC-Py^7^ to allow for direct comparison of apparent binding.

#### Direct DNA binding analysis

The binding affinity of Cu_2_-DC-PyA was analysed by monitoring fluorescence upon titration of ctDNA. In 96-well plate, samples were prepared to contain varying concentrations of ctDNA *via* serial dilution. 50 µL of a 20 µM Cu_2_-DC-PyA solution was then added. Plates were incubated at RT for 30 min before reading fluorescence intensity (excitation/emission: 415/455 nm). Final conditions: 100 µL volume, varying ctDNA concentration, 10 µM Cu_2_-DC-PyA, 80 mM HEPES, 25 mM NaCl, pH 7.4. Data was normalised to provide a plot of fraction bound *versus* ctDNA concentration and the resulting data was fit in GraphPad prism using eqn (2) and (3). Fig. S13–S16.2
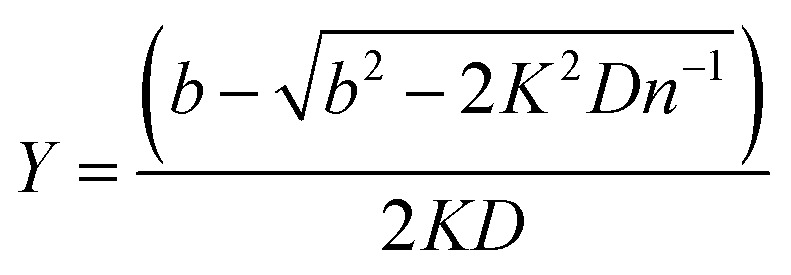
3
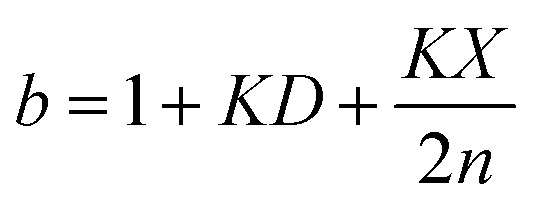
where *Y* is the fraction bound, *K* is the binding affinity, *D* the concentration of the target DNA (Cu_2_-DC-PyA), *N* is the binding site size or stoichiometry and *X* is the concentration of titrant (ctDNA).

#### Fluorescence resonance energy transfer (FRET) melting

Thermal melting analysis was performed on a Roche LightCycler®480 II using 80 mM HEPES 25 mM NaCl buffer. Prior to analysis DNA hairpins were denatured by heating to 90 °C (10 °C min^−1^, 2 min hold) and reannealed at 12 °C (0.5 °C min^−1^, 20 min hold). A serial dilution of Cu_2_-DC-PyA (highest concentration = 10 µM) was performed and FRET-labelled hairpin was added to a final concentration 1 µM. Melting was conducted in triplicate at a ramp rate of 0.5 °C min^−1^ up to a maximum of 95 °C. *T*_m_ values were calculated from the midpoint of the melting curves, which was found at the peak of the first derivative.

DDH – 5′-F-CGCGAATTCGCGAAAAACGCGAATTCGCG-Q-3′

D6aH – 5′-F-GCATTATAATGCAAAAAGCATTATAATGC-Q-3′

A schematic of the FRET melting analysis of the FRET hairpins can be seen in Fig. S17, where F = Alexa fluor 647 and Q = Iowa black quencher.

#### Topoisomerase IA relaxation

Topoisomerase IA (topo) relaxation was performed as previously reported with slight modification.^22^ In a total volume of 15 µL, pUC19 supercoiled plasmid DNA (400 ng) and increasing concentrations (0.25–500 µM) of drug (Cu_2_-DC-PyA, Cu_2_-DC-PyN_3_) were incubated in HEPES buffer (80 mM, pH 7.2) at 37 °C for 30 min. rCutsmart (1 ×, NEB) and topo IA (1 U, NEB) were added and samples were incubated at 37 °C for 20 min. The topo enzyme was heat inactivated at 65 °C for 30 min. DNA loading buffer (6×, 10 mM Tris-HCl, pH 7.6, 0.03%bromophenol blue, 0.03% xylene cyanol FF, 60% glycerol, 60 mM EDTA) was added and samples were loaded onto a 1.2% agarose gel (1× TBE) and run at 40 V for 3 h, followed by 50 V for 2.5 h. The gel was post-stained in SYBRsafe^TM^ (1×) for 20 min, followed by de-staining in deionised (DI) H_2_O for 20 min. Images were captured on a UV transilluminator.

#### Viscosity studies

To determine how various compounds effect the viscosity of DNA, a DV-II-Programmable Digital Viscometer equipped with Enhanced Brookfield UL Adapter was used. This method was carried out at 60 rpm using spindle #0 in working samples of 15 mL stDNA (deoxyribonucleic acid sodium salt from salmon testes, Sigma-Aldrich, D1626) prepared at 1 mM in 80 mM HEPES buffer. Stock solutions of each compound were titrated into the stDNA at increasing [drug]/[DNA] (*r*) ratios of 0.02, 0.04, 0.06, 0.08, 0.1, 0.12, 0.14, 0.16, 0.18, and 0.2. Titrations were carried out directly into the viscometer with constant stirring and left for 10 min before readings were taken, thus allowing for drug-DNA binding equilibrium to be reached. Data were then plotted using *η*/*η*_0_ against [drug]/[DNA] ratios, in which *η* refers to the cP value in the presence of drug, and *η*_0_ refers to the initial cP value without titrant.

#### DNA cleavage

In a total volume of 20 µL (80 mM HEPES, 25 mM NaCl) Cu_2_-DC-PyA or Cu_2_-DC-PyN_3_ (2.6–100 µM), pUC19 plasmid DNA (400 ng) and Na-l-ascorbate (1 mM) were incubated 37 °C for 30 minutes. Loading dye (6×, 10 mM Tris-HCl, 0.03% bromophenol blue, 0.03% xylene cyanole FF, 60% glycerol, 60 mM EDTA) was added and samples were loaded onto a 1.3% agarose gel (1× TAE) containing SYBRsafe^TM^. Gel was run at 70 V for 90 min prior to visualisation on a UV transilluminator.

#### Groove blocking

Samples were prepared as described above at Cu_2_-DC-Py-HA (1, 5, 10, 20 µM), but methyl green (8 µM), netropsin (8 µM), actinomycin-D (8 µM) or EtBr (8 µM) were added prior to addition of Cu_2_-DC-PyA. Samples were incubated at 37 °C for 30 min and analysed by agarose gel electrophoresis (1.3% agarose, 1× TAE, 70 V for 90 min). Loading dye (6×, 10 mM Tris-HCl, 0.03% bromophenol blue, 0.03% xylene cyanole FF, 60% glycerol, 60 mM EDTA) was added and gel was visualised.

#### Reactive oxygen species (ROS) scavengers

Samples were prepared as described above at Cu_2_-DC-PyA (1, 5, 10, 20 µM), but ROS scavengers (1 mM) were added prior to addition of Cu_2_-DC-PyA. Samples were incubated at 37 °C for 30 min and analysed by agarose gel electrophoresis (1.3% agarose, 1× TAE, 70 V for 90 min). Loading dye (6×, 10 mM Tris-HCl, 0.03% bromophenol blue, 0.03% xylene cyanole FF, 60% glycerol, 60 mM EDTA) was added and gel was visualised.

#### Real-time quantitative PCR (qPCR)

A 411bp amplicon of pUC19 (forward: 5′-TGACTCCCCGTCGTGTAGAT-3′ and reverse: 5′-TGATAACACTGCGGCCAACT-3′) was generated by PCR (PCR Biosystems) and purified using membrane spin columns (Monarch PCR & DNA clean-up kit, NEB). The 411 bp amplicon (200 ng) was diluted to 8 µL in HEPES (80 mM, pH 7.2). Half of the sample volume (4 µL) was removed and kept as reference samples. The remaining 4 µL incubated with increasing concentrations of Cu_2_-DC-PyA (0.25–10 µM) and Na-l-ascorbate (1 mM). Samples were incubated at 37 °C for 30 min and quenched with EDTA (1 mM). Diluted (1 : 4^3^) reference and experimental samples were analysed by qPCR through amplification of a 254 bp region (forward: 5′-CAGTGCTGCAATGATACCGC-3′ and reverse: 5′-GGGAACCGGAGCTGAATGAA-3′) over 45 cycles through SYBR green I fluorescence (SYBR green I master, Roche). The changes in *C*_T_ (threshold cycle) between experiment and reference samples (experiment *C*_T_–reference *C*_T_) were calculated (Δ*C*_T_). Δ*C*_T_ was then normalised to a non-treated control (Δ*C*_T_ sample − Δ*C*_T_ control = ΔΔ*C*_T_), and this was plotted as a linear value 2^−ΔΔ*C*_T_^. Ordinary one-way ANOVA with Dunnett's multiple comparisons was performed, using GraphPad Prism, to determine the significance of the 2−ΔΔ*C* values obtained. * = *P* = <0.05, significant ** = *P* < 0.01, very significant. *** = *P* < 0.001, extremely significant.

## Author contributions

OG: formal analysis, writing (original content, reviewing and editing), AG: funding acquisition, formal analysis, writing (original content, review and editing), conceptualisation, supervision, project administration, methodology. RL: formal analysis. SP: formal analysis, supervision, conceptualisation, writing (review and editing). BMcG: funding acquisition, formal analysis, writing (original content, review and editing), methodology, supervision, project administration. AK: funding acquisition, writing (review and editing), supervision, project administration, conceptualisation.

## Conflicts of interest

There are no conflicts to declare.

## Supplementary Material

CB-OLF-D6CB00097E-s001

## Data Availability

All data supporting the findings of this study are available within the article and its supplementary information (SI). Supplementary information: includes NMR spectra, FT-IR spectra, LC-MS data, fluorescence binding data, FRET melting curves, Viscosity data, and gel electrophoresis images supporting the synthesis, characterisation, and biological assays described in the manuscript respectively. Additional raw datasets generated or analysed during the current study are available from the corresponding authors upon reasonable request. See DOI: https://doi.org/10.1039/d6cb00097e.
