# Supplementary material for: Rational design of an acridine-derived click chemistry-based artificial metallo-nuclease
Source: RSC Chem Biol. 2026 Jun 15. Online ahead of print. doi: 10.1039/d6cb00097e (PMC13326999; doi:10.1039/d6cb00097e)
Supplement: CB-OLF-D6CB00097E-s001 [file CB-OLF-D6CB00097E-s001.pdf]

Supplementary Information (SI) for RSC Chemical Biology.
This journal is © The Royal Society of Chemistry 2026

Rational Design of an Acridine-derived Click Chemistry-based Artificial Metallo-Nuclease

Oliver Gould,† Alex Gibney,† Rebecca Lynn, Simon Poole, Bríonna McGorman,* and Andrew Kellett*

School of Chemical Sciences, Dublin City University, Glasnevin, Dublin 9, Dublin, Ireland.

† These authors contributed equally.
andrew.kellett@dcu.ie

Supplementary Figures and Data

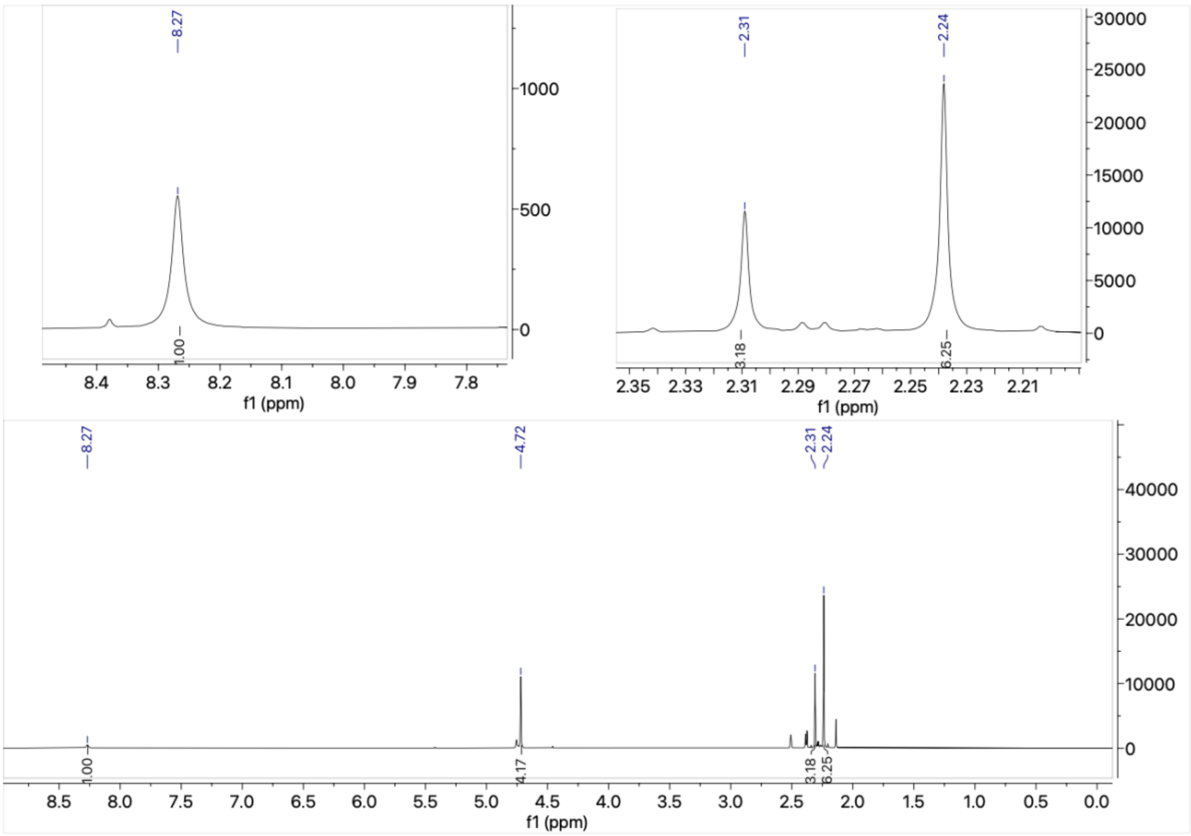

Figure S1: ^{1}H NMR spectrum of dibromide (**1**) in DMSO-d_6

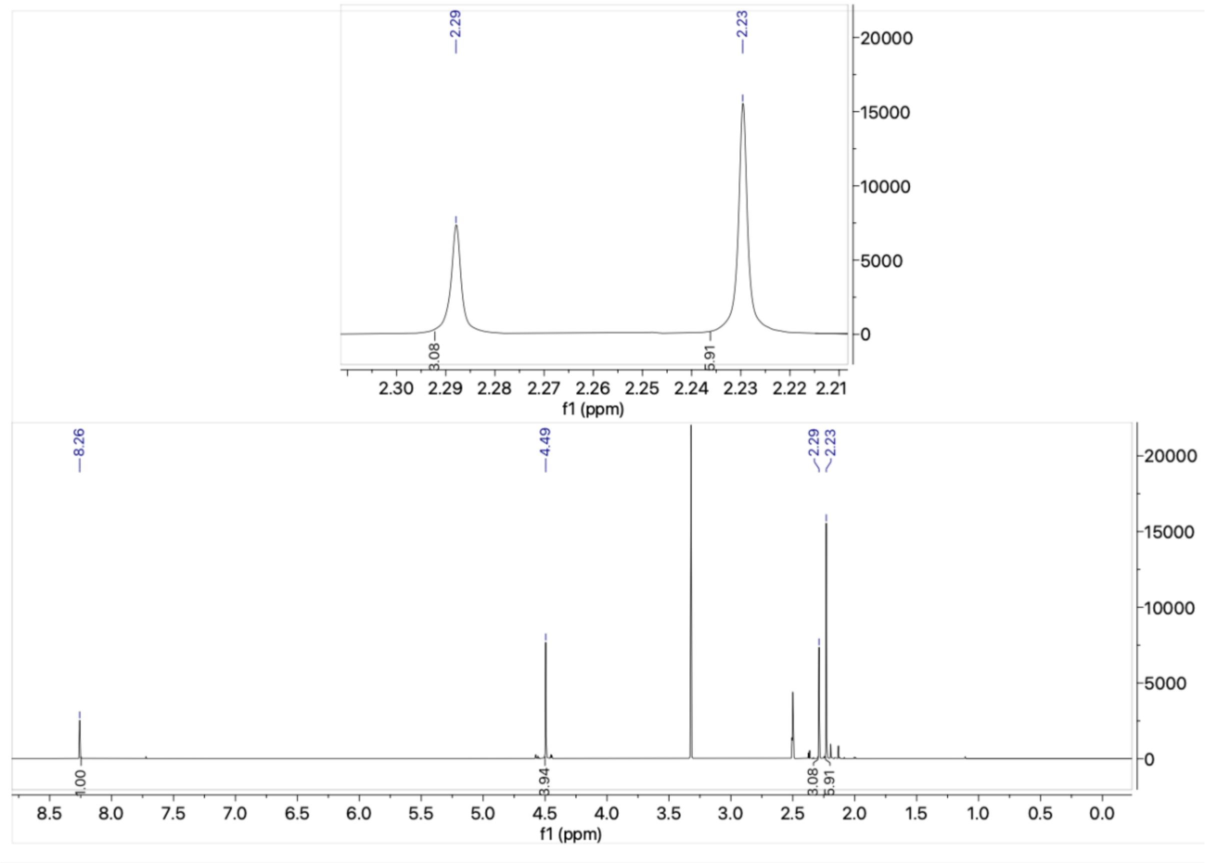

Figure S2: ^{1}H NMR spectrum of diazide (**2**) in DMSO-d_6

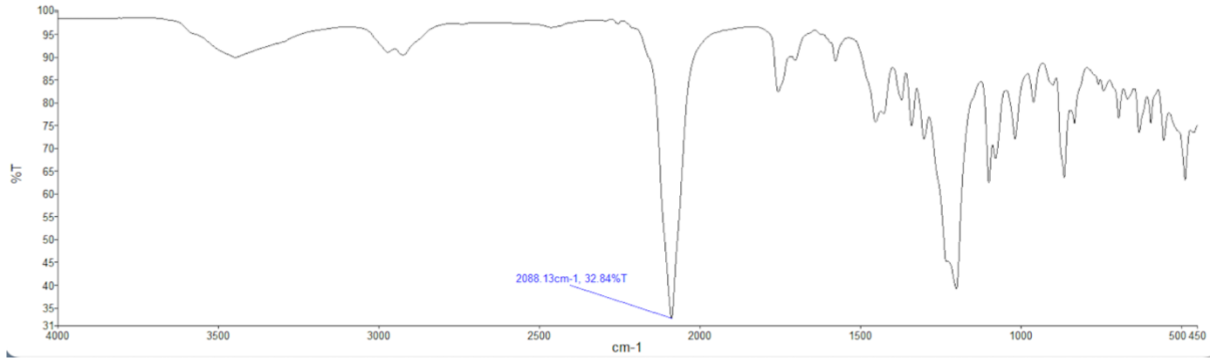

Figure S3: FTIR spectrum of 2 showing azide peak at 2088 cm^{-1}

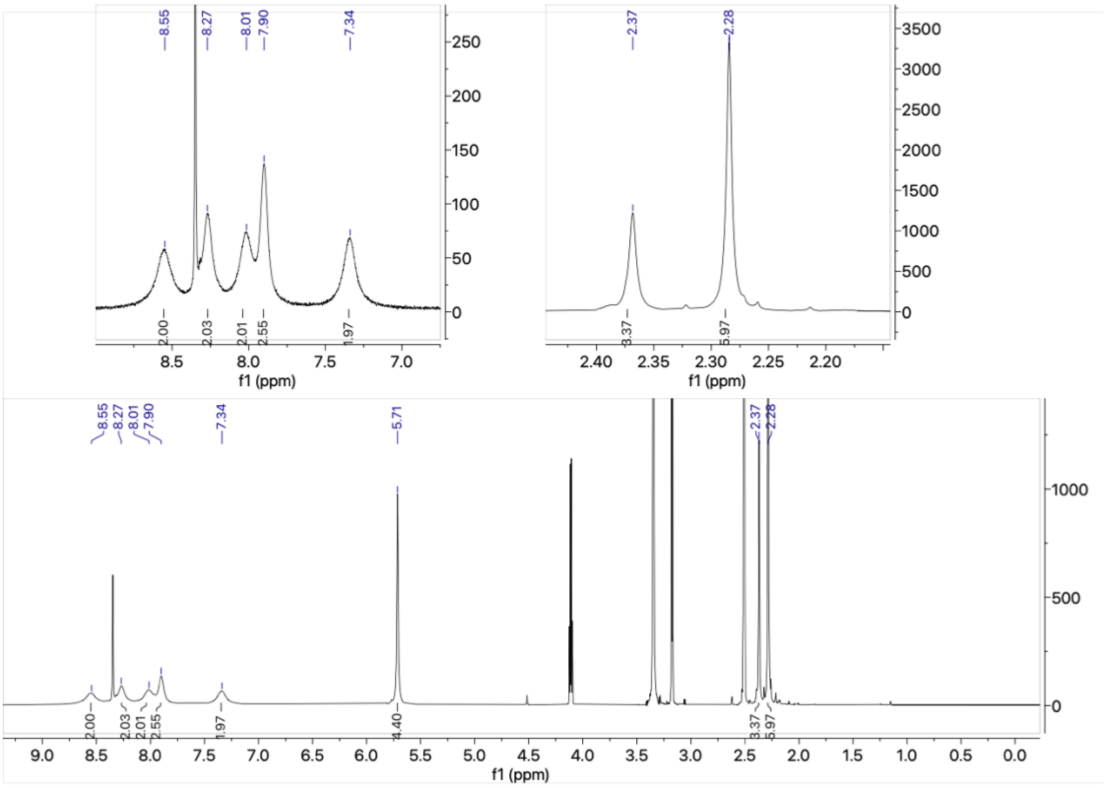

Figure S4: ^{1}H NMR spectrum of DC-Py (**3**) in DMSO-d_6

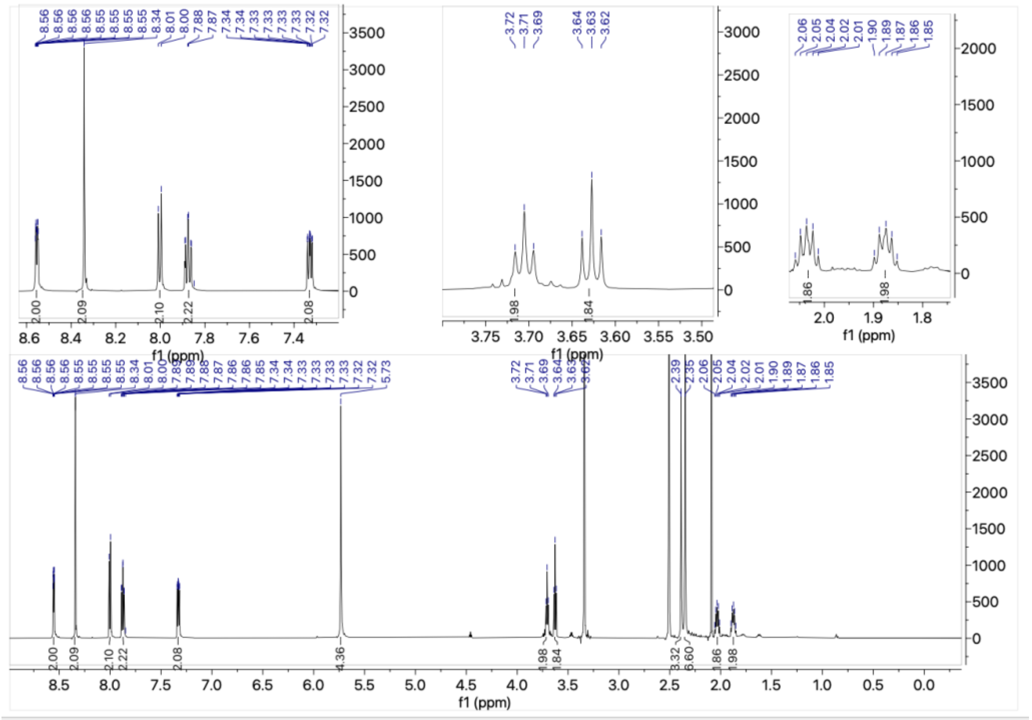

Figure S5: ^{1}H NMR spectrum of DC-PyBr (**4**) in DMSO-d_6

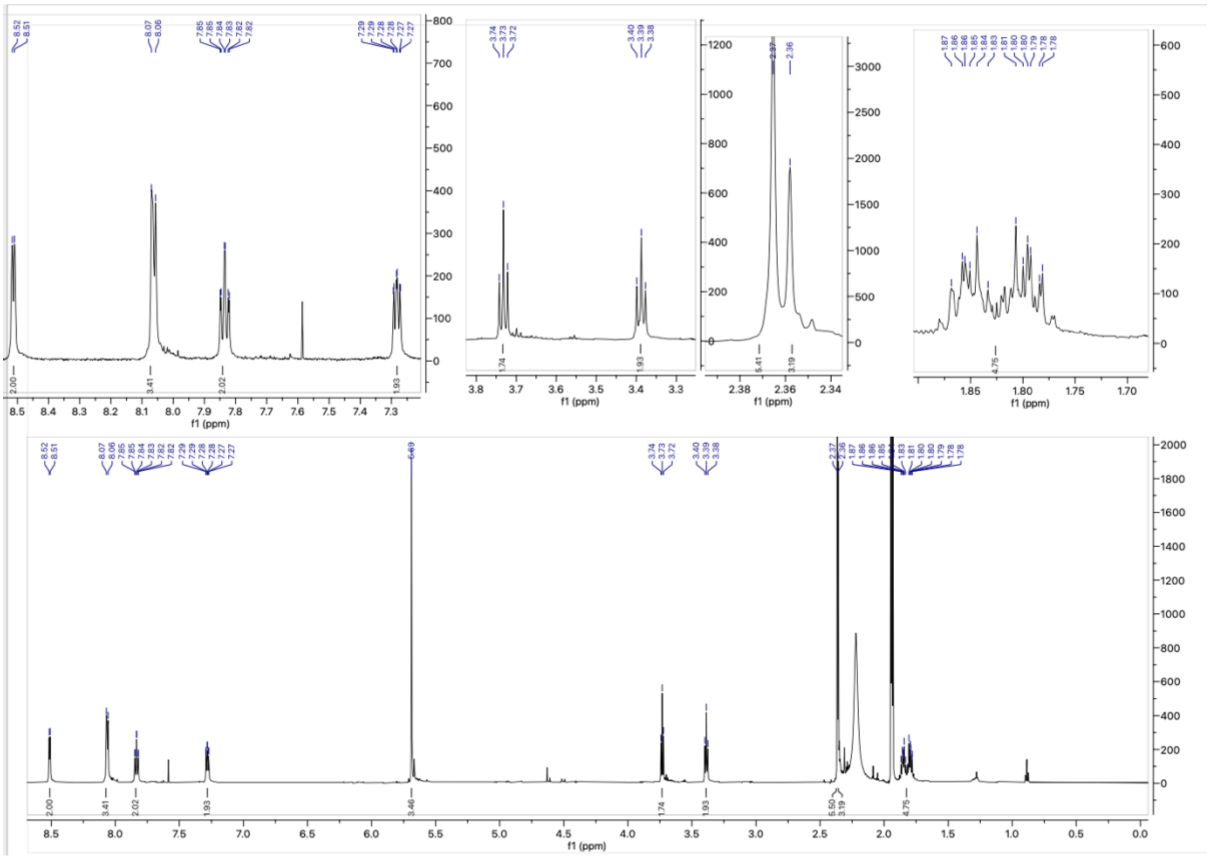

Figure S6: ^{1}H NMR spectrum of DC-PyN$_3$ (**5**) in DMSO-d$_6$

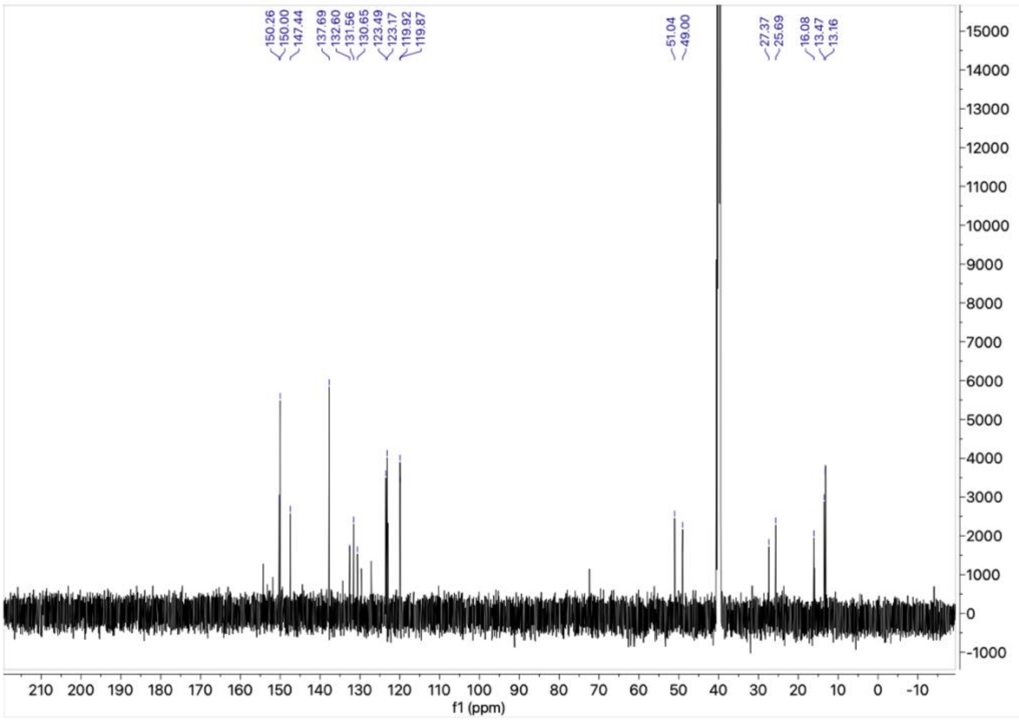

Figure S7: ^{13}C NMR spectrum of DC-PyN$_3$ (**5**) in DMSO-d$_6$

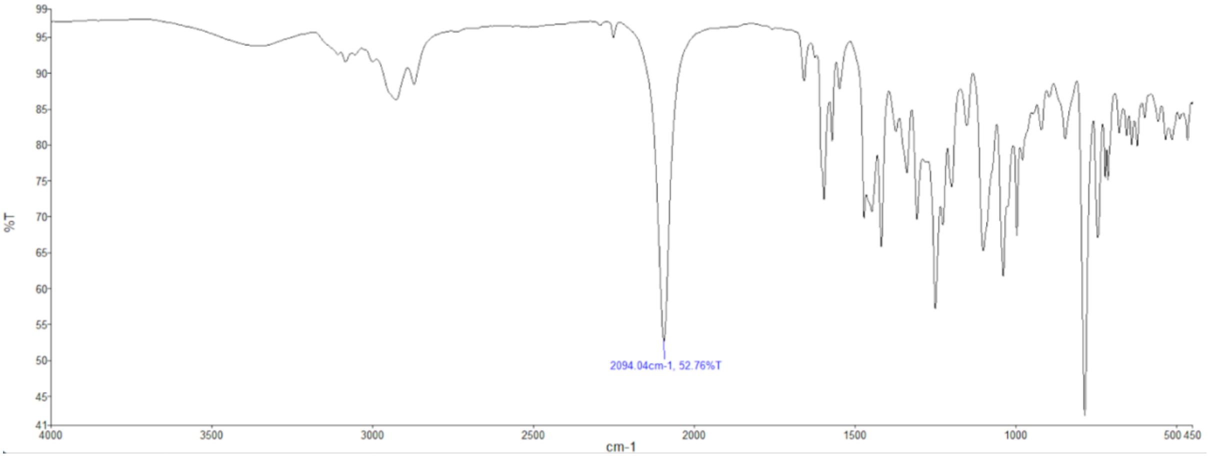

Figure S8: FTIR spectrum of 5, showing azide signal at 2094 cm^{-1}.

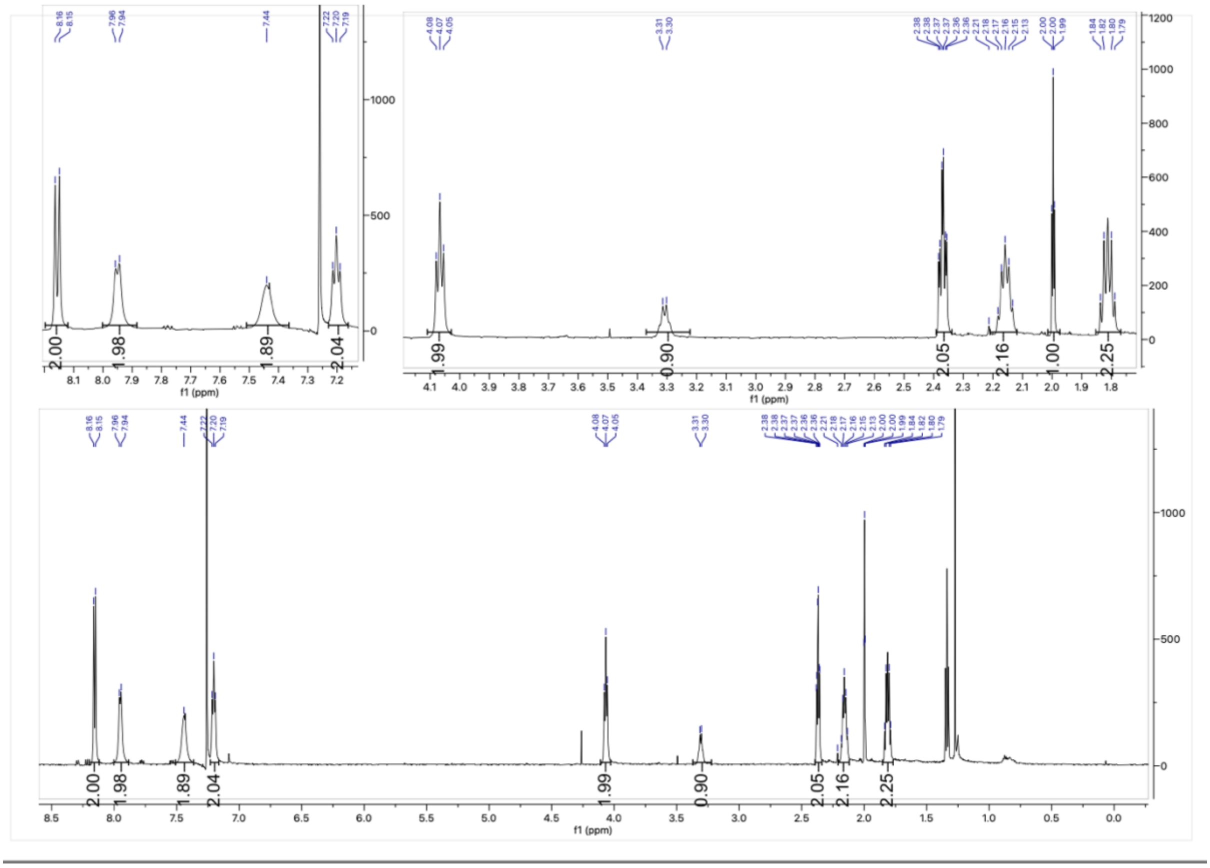

Figure S9: ^{1}H NMR spectrum of Hexynyl acridine (**6**) in $CDCl_3$.

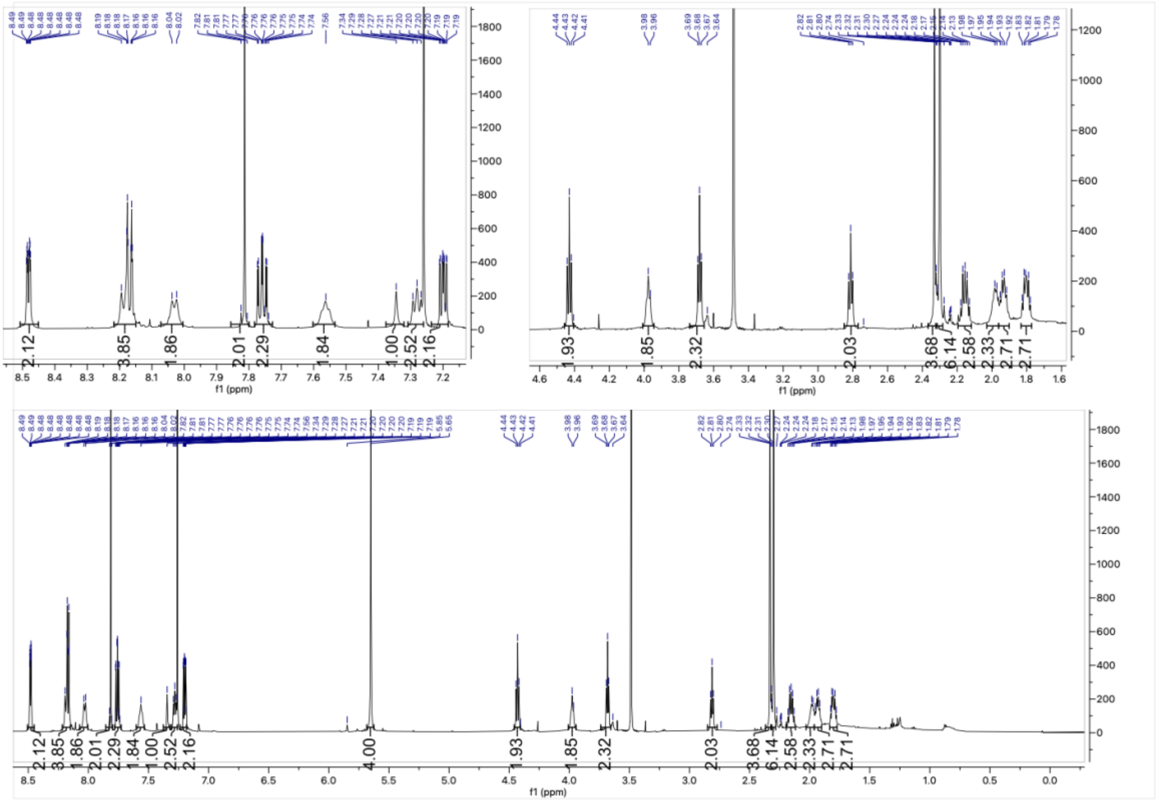

Figure S10: ^{1}H NMR spectrum of DC-PyA (**7**) in $CDCl_3$.

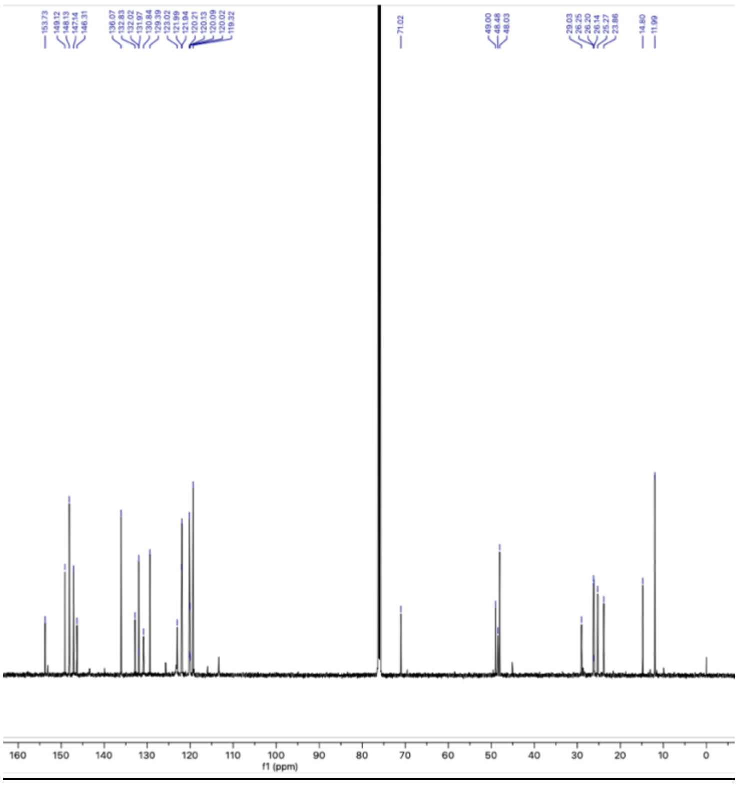

Figure S11: ^{13}C NMR spectrum of DC-PyA (**7**) in $CDCl_3$.

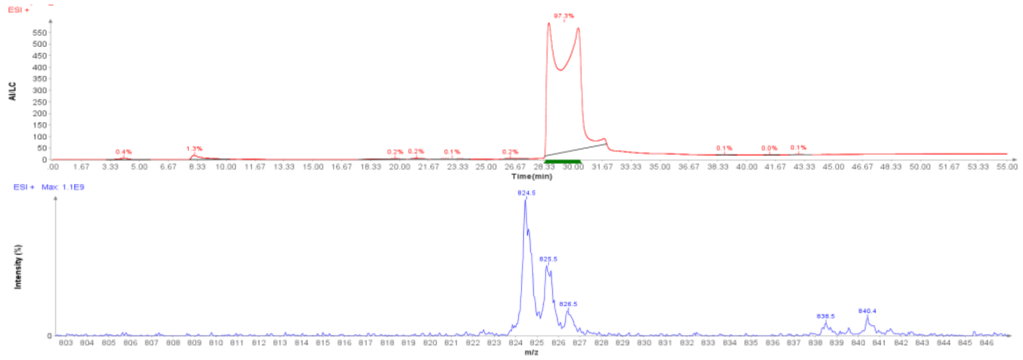

Figure S12: LC-MS and ESI-MS of DC-PyA (**7**).

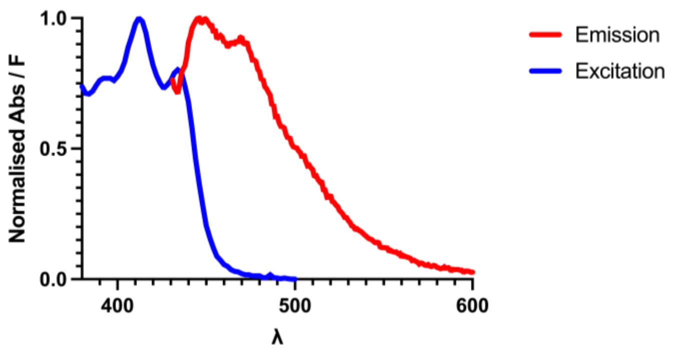

Figure S13: Excitation and emission scans of Cu_2-DC-PyA in 80mM HEPES, 25mM NaCl.

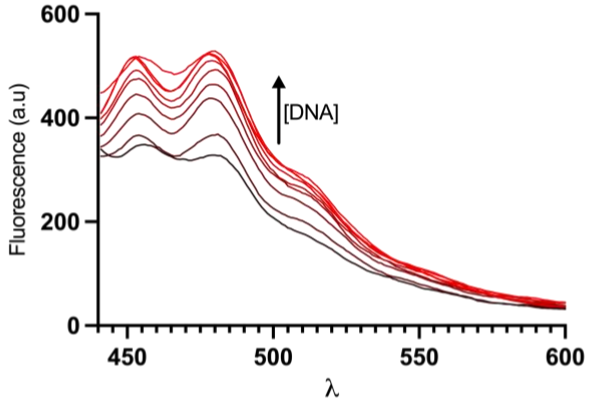

Figure S14: Fluorescence scans with a fixed (10 µM) concentration of Cu_2-DC-PyA and increasing ctDNA concentrations of ctDNA (excitation 415 nm).

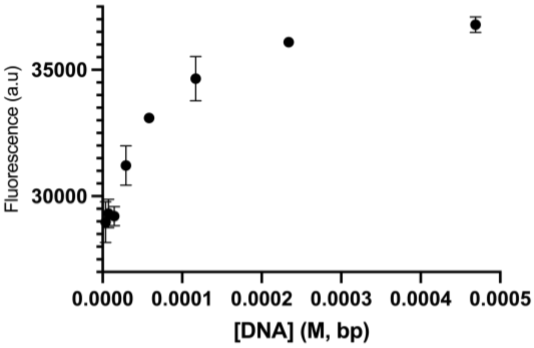

Figure S15: Raw fluorescence values of 10 µM Cu_2-DC-PyA with increasing ctDNA concentrations (excitation 415 nm, emission 455 nm).

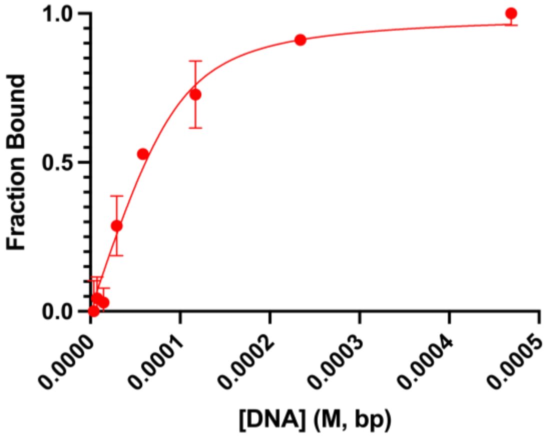

Figure S16: Binding curve and Bard fit of fraction bound of Cu_2-DC-PyA bound vs ctDNA concentration. Bard model was fit using equations S1 and S2.

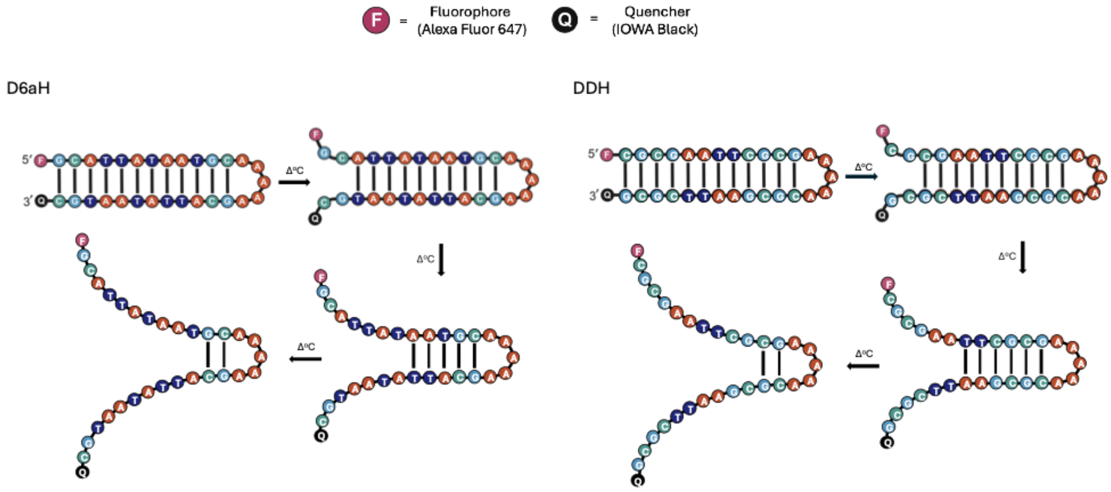

Figure S17: Cartoon representation of FRET melting using DNA hairpins DDH and D6aH.

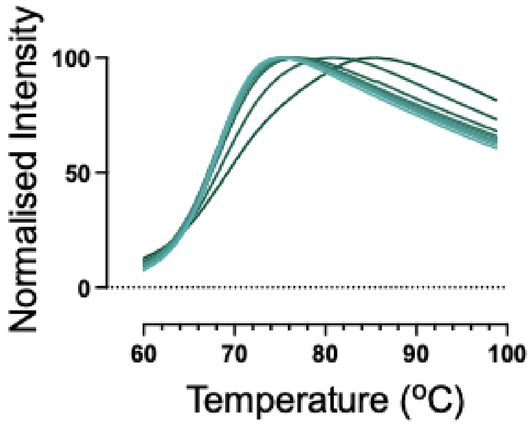

Figure S18: FRET melting curve of D6aH with Cu_2-DC-PyA.

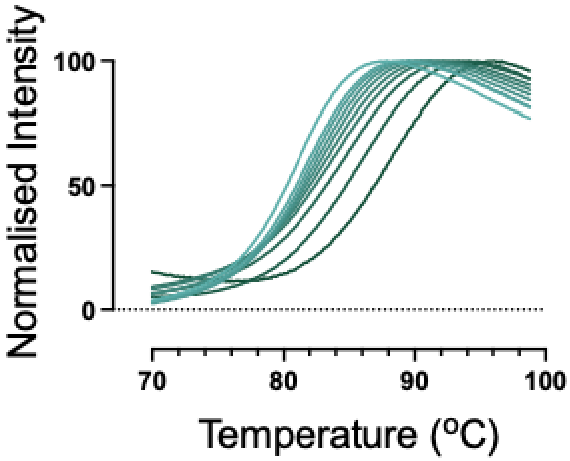

Figure S19: FRET melting curves of DDH with Cu_2-DC-PyA.

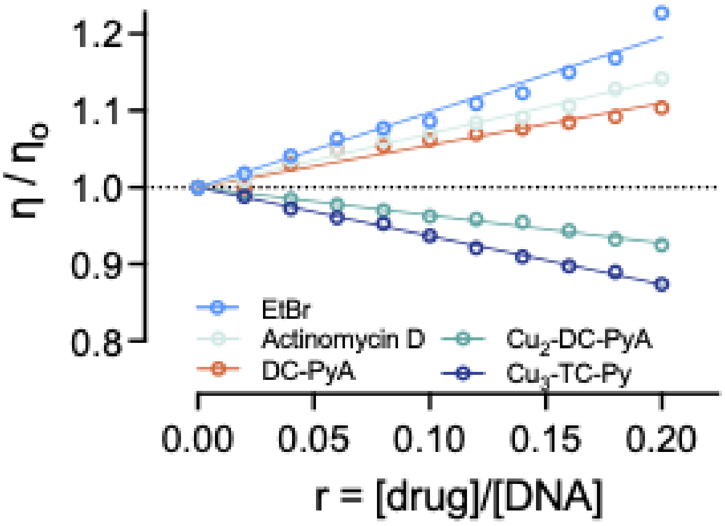

Figure S20: Plot of relative viscosity versus drug loading ratio for control and test compounds.

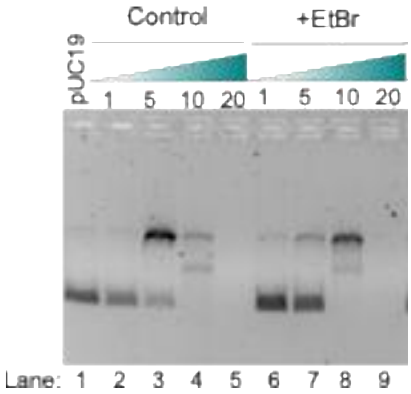

Figure S21. pUC19 cleavage analysis via EMSA, comparing a control concentration ramp containing 1, 5 , 10 and 20 μM Cu2-DC-PyA (lanes 2-5) to the same experiment in the presence of 8 μM EtBr.
